# Osteonecrosis of the Femoral Head

**DOI:** 10.5435/JAAOSGlobal-D-21-00176

**Published:** 2022-05-05

**Authors:** Gary George, Joseph M. Lane

**Affiliations:** From Weill Cornell Medicine, New York, New York (Mr. George), and the Hospital for Special Surgery, New York, New York (Dr. Lane).

## Abstract

Osteonecrosis of the femoral head is a progressive and debilitating condition with a wide variety of etiologies including trauma, steroid use, and alcohol intake. Diagnosis and staging are based on imaging including MRI at any stage and plain radiography in more advanced lesions. The only definitive treatment is total hip arthroplasty, although numerous treatments including disphosphonates and core decompression are used to delay the progression. Lack of satisfactory conservative measures suggests the need for additional research of osteonecrosis including large patient registries to further understand this condition.

Osteonecrosis is a progressive disorder in which lack of sufficient blood supply leads to cell death, fracture, and collapse of the affected area. The condition is frequently associated with the femoral head, where progression can be debilitating and can ultimately necessitate total hip arthroplasty (THA). The etiology of osteonecrosis is complex with numerous contributing agents, most markedly trauma, steroid use, and alcohol. Treatment of osteonecrosis is controversial because no option has been overwhelmingly embraced, and little research has compared treatments. Researchers estimate that 20,000 new cases of osteonecrosis are diagnosed in the United States each year.^1^ The increasing incidence and debilitating progression of osteonecrosis suggest the need for additional investigation of effective and novel treatments, as well as the need for clearer understanding of available treatments. This review characterizes the current knowledge on etiology, pathophysiology, epidemiology, and clinical management of osteonecrosis, with an emphasis on recent developments.

## Epidemiology

The incidence of osteonecrosis in the United States has been estimated at ∼20000 to 30000 cases per year, affecting primarily young adults between the ages of 20 to 40 years.^1^ Recent analysis has shown that although the number of THAs done for osteonecrosis has increased between 2001 and 2010 (from 54.2 per 100,000 hospital admission to 60.6 per 100,000 hospital admission), the percentage of THAs done for osteonecrosis has decreased from 9.7% to 8.3%, likely because of the rapid increase in osteoarthritis necessitating THA.^2^

## Pathophysiology and Pathogenesis

### General Pathogenesis

Osteonecrosis occurs because of compromised blood flow or oxygen delivery to the bone, although the clinical presentation is a result of the repair process, rather than initial ischemia. In osteonecrosis, bone formation by osteoblasts is unable to match bone resorption by osteoclasts. This remodeling imbalance does not adequately replace the necrotic bone, leaving a region of structurally unsound bone tissues.^3^

### Trauma

Trauma is the most common cause of osteonecrosis,^4^ disrupting blood flow and leading to osteocyte death. Estimates of occurrence of traumatic osteonecrosis of the femoral head vary depending on the injury type^5^; however, in meta-analysis of traumatic osteonecrosis, incidence has been found to be as high as 14.3%.^6^ The Garden classification categorizes femoral neck fractures and can be used to estimate the risk of osteonecrosis. Garden I (incomplete fracture) and Garden II (complete and nondisplaced) are considered stable and low risk, and can be repaired with internal fixation. Garden III (complete and partially displaced) and Garden IV (complete and completely displaced) have much higher rates of osteonecrosis with internal fixation (16%), and arthroplasty should be considered.^7^ Intertrochanteric hip fractures result in a low risk of osteonecrosis, noted at 0.95% after a 1-year follow-up.^8^

### Atraumatic Osteonecrosis

Atraumatic osteonecrosis encompasses a diverse array of causes. It is important to note that in atraumatic osteonecrosis, disease is frequently bilateral owing to systemic risk factors, with some estimates suggesting as high as 70% of the patients with unilateral osteonecrosis developing disease in the contralateral hip.^9,10^ The reasons for the sparing of one hip in the presence of a systemic risk factor are not well studied and may be because of subclinical presentation, differences in wear patterns between hips, underinvestigation of symptoms, or lack of coordinated follow-up.

### Glucocorticoids

Steroid use is the second most common cause of osteonecrosis.^11,12^ Several potential mechanisms have been proposed for this association, including bone matrix and cartilage degeneration, induced stem cell abnormalities, changes in lipid metabolism, creation of fat emboli, altered coagulation, and changes in blood supply.^11,12^ Meta-analysis found up to 10 times increased risk of patients on high-dose corticosteroids, a doubling of risk for osteonecrosis when the cumulative dose exceeds 10 g, and a 0% increase in risk with each 10 mg increase of daily dose.^13^ Corticosteroids have also been implicated in osteoblast death and decreased osteoblast proliferation, impairing the ability to repair and replace necrotic lesions.^11^

### Alcohol

Alcohol is hypothesized to act through altered lipid metabolism and increased adipogenesis.^14^ It is hypothesized that increased generation of lipids increases the risk for fat emboli leading to vascular occlusion. In addition, increased serum lipids can cause packing of the marrow, increasing intraosseous pressure and decreasing blood flow.^5,12^ Alcohol may also contribute to osteocyte death.^5^ A study has also shown increased cortisol levels in patients with alcohol-induced osteonecrosis compared with idiopathic osteonecrosis control subjects, suggesting that alcohol-induced osteonecrosis may act through the steroid pathway.^15^ Previous estimates noted an 11 times higher risk of osteonecrosis in consumers of >400 mL of alcohol daily.^16^

### Hyperlipidemia

Hyperlipidemia is thought to decrease the blood supply to affected regions by increasing intraosseous pressure and producing fat emboli.^4^ One study of low-energy femoral neck fractures in the elderly found higher blood lipid abnormalities in those who developed osteonecrosis than those who did not.^17^ A study of patients with acute lymphoblastic leukemia (ALL) identified hyperlipidemia as a risk factor for developing osteonecrosis.^18^ A similar study found association with osteonecrosis development in patients with hyperlipidemia and systemic lupus erythematosus (SLE).^19^

### Systemic Lupus Erythematosus

The association of SLE with osteonecrosis is related to frequent corticosteroid treatment; however, recent analysis has shown higher incidence of osteonecrosis in corticosteroid users with SLE than in corticosteroid users without SLE, suggesting synergistic effects.^20^ Meta-analysis of SLE studies has identified numerous noncorticosteroid risk factors in SLE, notably renal involvement and central nervous system (CNS) disease.^21,22^ Mixed data suggest that the prothrombotic effects of antiphospholipid antibodies play a role in osteonecrosis development in SLE. Recent meta-analysis of childhood-onset SLE found notable osteonecrosis association, with estimates that 6 to 8.4% of the patients with childhood-onset SLE develop osteonecrosis,^23^ although most did not develop osteonecrosis until after puberty.^21^

### Sickle Cell Disease

Studies of the association between sickle cell disease and osteonecrosis have identified 2 to 4.5 cases of osteonecrosis per 100 patients with sickle cell disease.^24^ Precipitation of hemoglobin S in low-oxygen environments may lead to vaso-occlusion and ischemia of the bone, which is similar to the development of other vaso-occlusive injury in sickle cell disease.^5^ A recent study supports this theory, citing elevated hemoglobin levels as a risk factor for osteonecrosis in patients with sickle cell disease and suggesting that vaso-occlusion, high blood viscosity, hypoxia, and concurrent alpha-thalassemia contribute to osteonecrosis.^25^

### Gaucher Disease

A recent evaluation of the Gaucher Registry estimated the incidence of osteonecrosis at 30%.^26^ Gaucher disease may act through a similar path to that of sickle cell disease, with Gaucher-affected cells obstructing the blood flow^27^ or by increasing intraosseous pressure because they accumulate in the fatty marrow.^3^ In addition, Gaucher cells can release osteoclast-activating cytokines which disrupt the balance of bone formation and resorption.^26^ Enzyme replacement can reduce or delay the symptoms of osteonecrosis^28^; however, a study has suggested that the bone marrow may serve as a “sanctuary site” for Gaucher cells, leaving a subset of patients vulnerable to osteonecrosis despite treatment.^29^

### Decompression Sickness

Decompression sickness–related osteonecrosis or dysbaric osteonecrosis occurs because of rapid decompression after an extended period in a hyperbaric environment. Rapid decompression forms bubbles in the bloodstream because dissolved nitrogen comes out of the solution. The high solubility of nitrogen in fatty tissues makes the marrow particularly susceptible. Multiple mechanisms have been proposed, including direct occlusion of blood flow to the marrow and the increase in intraosseous pressure reducing effective blood flow.^30^ A recent study of divers with musculoskeletal decompression sickness found evidence of dysbaric osteonecrosis in 26% of the cases, although the study was limited by the relative rarity of this condition.^31^

### Acute Lymphoblastic Leukemia

Patients with ALL show an increased risk of osteonecrosis, with radiographic incidence reaching 71.8% in prospective studies.^32^ The single largest factor identified in the development of osteonecrosis in patients with ALL is adolescence, suggesting an effect of ALL or its treatment on the growth and remodeling of the bone. It is also possible that the occurrence of this time of changing metabolism and growth magnifies susceptibility to osteonecrosis-causing damage from other factors.^33^ Older adults, who make up a small portion of those diagnosed with osteonecrosis, often undergo modified treatment regimens and have worse overall outcomes compared with their younger counterparts.^34^ A recent study of childhood leukemias found higher incidence of osteonecrosis in patients treated with hematopoietic stem cell transplant (HSCT) versus chemotherapy alone (6.8% versus 1.4%), suggesting that treatment methods influence osteonecrosis development.^35^ In addition, a review of treatment regimens identified increased cumulative dose of steroids as a risk factor for developing osteonecrosis in children with any hematologic malignancy.^36^ A review of treatment strategies suggested that the use of discontinuous steroid regimens may decrease the risk of osteonecrosis and nonsteroid chemotherapeutic agents such as methotrexate and asparaginase may contribute to the development of osteonecrosis.^37^ One trial of alternate week dexamethasone reduced the risk of osteonecrosis compared with continuous treatment in children with high-risk ALL.^38^

### Transplantation

A recent study suggests steroid-mediated development of osteonecrosis in transplant patients, finding cumulative steroid doses to be higher in renal transplant patients who developed osteonecrosis than in those who did not. The study also found that the incidence of symptomatic osteonecrosis decreased from 20% to less than 5% with the introduction of cyclosporine and a decrease in steroid usage.^39^

### HIV

Multiple studies show a growing incidence of osteonecrosis in patients with HIV, showing nearly three times the risk of the general population.^40^ One recent study revealed a strong association between high-activity antiretroviral therapy and development of osteonecrosis, although the authors caution that the association does not imply a pathologic role.^40^ Other studies have found no association between osteonecrosis and antiretroviral therapy (ART), citing instead association with alcohol, hyperlipidemia,^41^ or low nadir CD4 counts,^42^ although the mechanism is not well understood.

### Genetic Involvement

Although familial variants of osteonecrosis and some associated genes have been found, no single responsible gene has been identified. One gene candidate is a mutation in type II collagen, although no definitive causality has been established.^43^ Elevated levels of osteoprotegerin and decreased expression of RANK/RANK ligand have been found in necrotic regions compared with healthy bone, suggesting a potential role of osteoclast-regulating genes.^44^ Factor V Leiden mutations and prothrombin mutations have been associated with patients with osteonecrosis in multiple studies,^43^ invoking a potential role of altered coagulation. Genome-wide association studies of selected populations have identified several loci of interest, including clusters of variants near glutamate receptor genes in patients with ALL,^45^ corticosteroid-induced osteonecrosis,^33^ and several loci of unknown significance, which may be related to coagulation pathways, lipid metabolism, or alcohol drinking behavior.^46^

### Idiopathic Osteonecrosis

It is important to note that an estimated 20% to 40% of osteonecrosis cases are idiopathic.^47^ This high rate of an unknown cause may be due to nonspecific early symptoms and indolent course, which prevent early diagnosis,^9^ as well as lack of standardized reporting and data collection, which may help to reveal little understood causes and connections.

## Clinical Manifestations and Diagnosis

### Diagnosis

The early stages of osteonecrosis of the femoral head are frequently asymptomatic but may also present with radiating pain from the hip or groin and limited range of motion of the joint on physical examination.^47^ Diagnosis of osteonecrosis is primarily based on imaging, although examination and history are important to gather surrounding context and potential etiology.^5^ A plain radiograph is an appropriate first-line modality for identifying cases of osteonecrosis, with benefits including low cost, high availability, and adequate sensitivity for mid-stage and late-stage disease.^48^ Frontal and lateral “frog-leg” views are recommended for accuracy. In the case of early-stage disease, radiography may be insufficient to identify early or minimal changes. MRI is the benchmark for diagnosis of osteonecrosis because of its high sensitivity for early signs of onset. Supplemental imaging, including diffusion-weighted MRI^49^ and gadolinium-enhanced perfusion MRI,^50,51^ may further advance the diagnostic capabilities of MRI. Perfusion MRI may assist in distinguishing between radiographically and symptomatically similar conditions such as bone marrow edema and subchondral insufficiency fractures.^52^ In pediatric patients with developmental dysplasia of the hip, perfusion MRI was helpful in identifying those at risk for osteonecrosis after closed reduction/spica casting.^53^ In addition, a whole-body bone scan provides an option for patients at risk for multifocal osteonecrosis, such as those receiving systemic corticosteroids or immunosuppressants.^54^

### Differential Diagnosis

#### Bone Marrow Edema Syndrome

Bone marrow edema syndrome (BMES) presents as sudden pain without a clear precipitating event. On imaging, BMES shows diffuse edema compared with more localized areas in osteonecrosis. Some studies have suggested that BMES may precede osteonecrosis.^5^

#### Subchondral Insufficiency Fracture

A subchondral insufficiency fracture presents similarly but occurs after an injury. Although both conditions present with low-signal subchondral bands, osteonecrosis imaging presents with a smooth, concave line while the fracture presents with a jagged, discontinuous, convex finding. Conservative treatment is unlikely to improve fracture symptoms, and both conditions can progress to the need for a THA.^5^

#### Neoplasm

Although rare, clear cell chondrosarcoma and chondroblastoma can present with radiolucent lesions in the femoral head. These conditions are not accompanied by the edema present in osteonecrosis or other similar conditions such as BMES.^5^

## Classification Systems and Staging

The most popular staging system for osteonecrosis of the femoral head is the Ficat classification (Table 1). Developed in 1964 and later modified to include the use of MRI, the Ficat system classifies patients with osteonecrosis as stage 0 to 4 based on the appearance on a plain radiograph. Although this system is widely accepted and frequently used, detractors cite the use of clinical symptoms, low interobserver consensus, and lack of prognostication as limitations.^55^ The University of Pennsylvania system was developed in an attempt to more clearly delineate the progression of osteonecrosis and to promote distinctions between the stages by adding stage 0 for preradiographic disease, dividing Ficat stage II into two stages based on the absence (II) or presence (III) of a crescent sign, and dividing Ficat IV into two stages: flattening with joint space narrowing only (V) and joint deformity and joint space obliteration (VI).^56^ The Association Research Circulation Osseous (ARCO) system closely follows Ficat with the exception of the inclusion of MRI findings in stage I and division of stage II based on the extent of femoral head flattening (IIIA if < 2 mm and IIIB if > 2 mm). The ARCO system was recently revised based on an international expert taskforce to better incorporate results of both MRI and plain radiography.^57^ These staging systems are summarized in Table 1.

**Table 1:** Classification Systems of Osteonecrosis of the Femoral Head

	Hip		
	Stage	ARCO	UPenn
0			No radiographic evidence, but suspicion or clinical symptoms
I	Pain but no radiographic anomalies	Normal radiograph, abnormal MRI, or bone scan	Suggestive MRI, but a normal plain radiograph
II	Increased density, cystic changes, or porosity	Abnormal radiograph without fracture or flattening	Lucency, sclerosis, or plain radiograph and MRI
III	Flattening of the femoral head and crescent sign	Subchondral or necrotic zone fracture. IIIA: ≤2 mm flattening IIIB: >2 mm flattening	Crescent sign without flattening
IV	Full collapse of the femoral head with decrease in joint space	Radiographic evidence of arthritis with joint space narrowing, acetabular changes, and/or joint destruction	Flattening of the femoral head without joint space narrowing
V			Flattening of the femoral head with joint space narrowing
VI			Joint deformity and joint space obliteration

ARCO = Association Research Circulation Osseous

Systematic analysis of different staging systems found that any classification system is valuable and sufficient for the staging of osteonecrosis, provided necessary data are collected to allow conversion to another metric.^58^ For the purposes of patient evaluation and treatment, the most important classification is precollapse versus collapse because this guides discussion of conservative treatment versus THA. For research purposes (especially for the collection of registry data), we recommend using the updated ARCO guidelines because they effectively use multiple imaging modalities and delineate smaller changes between stages. This allows for a higher level of detail in tracking disease progression and may help to provide clearer answers because the effectiveness of new therapies is evaluated.

## Treatment Options

### Risk of Progression

Evaluating risk of progression is important in determining an appropriate treatment choice (Table 2). Although there is no consensus on a system to definitively predict collapse, a review of attempted strategies has found increased lesion volume, necrosis > 40% of the weight-bearing surface, and necrosis radian > 200 to 250 to be suggestive of future collapse.^59^

**Table 2:** Treatment Considerations Based on Lesion Collapse and Staging

Radiographic Presentation	Ficat Stages	ARCO Stages	UPenn Stages	Treatment Considerations
Precollapse	I II	I II	0 I II III	Risk factor modification when possiblePercutaneous drillingCore decompression±Stem cell augmentationBone graftingOsteotomy (location-based)
Collapse	III IV	IIIA IIIB IV	IV V VI	Bone graftingTotal hip arthroplasty (definitive)

ARCO = Association Research Circulation Osseous

### Observation

The most conservative management, observation, has been considered as a possible approach to osteonecrosis. There has been some evidence for spontaneous resolution of small early-stage osteonecrosis lesions.^60^ In combination with observation, restricted weight-bearing is usually advised, although this has not shown utility as a primary treatment modality.^61^ A study of observation as a strategy in osteonecrosis of the hip has found a failure rate of over 80% by four years and is not recommended as a standalone treatment in advanced lesions.^62^

### Nonsurgical Treatment

#### Pharmacologic Agents

Medications have been a mainstay of osteonecrosis treatment, but recently, their effectiveness has been questioned. Disphosphonates are a popular choice for pharmacologic treatment and work by inhibiting osteoclast activity. Studies of the use of disphosphonates have shown mixed results.^63^ Although some early studies showed positive effects of disphosphonates, a recent large multicenter randomized controlled trial found no difference between alendronate and placebo.^64^ Furthermore, a meta-analysis of five randomized controlled trials had similar findings, with little to no evidence supporting the efficacy of disphosphonates in the nontraumatic osteonecrosis of the femoral head.^65^ The primary utility of disphosphonates is in the early stages of disease, and they are not preferred to surgery as osteonecrosis progresses.^61^

Studies have identified multiple potential mechanisms for beneficial effects of statins in delaying osteonecrosis including lipid-lowering effects,^47^ increased autophagy,^66^ suppression of Peroxisome proliferator-activated receptorγ, and activation of the Wnt signaling pathway.^67^ Statins have been effective in combination with multiple core decompression (CD) procedures, improving both clinical and radiographic progression of osteonecrosis.^68^

#### Other Nonsurgical Modalities

Several other modalities have been proposed for the treatment of osteonecrosis with varying success. Lipid modifiers such as dietary changes or lipoic acid supplements have shown some positive results in trials, but there is insufficient evidence to recommend them as primary treatment strategies.^61,63^ Hyperbaric oxygen treatments, pulsed electromagnetic fields, and extracorporeal shockwave therapy have been proposed showing some positive outcomes, but disagreement about their effectiveness makes them difficult to recommend.^47,61,69^

### Joint Preserving Procedures

#### Core Decompression

CD is done for osteonecrosis of the femoral head to reduce intraosseous pressure and promote increased blood flow and bone genesis. Ficat,^70^ in his early descriptions of osteonecrosis and the CD procedure, noted increased intramedullary pressures, which are released with CD leading to a relief of pain and eventual restoration of blood flow if the lesion is treated early in its progression. Although older studies of CD were equivocal about its effectiveness, study of more recent procedures has shown notable benefits. Studies of both short-term and long-term outcomes have shown improvement in patients treated with CD and delayed time to THA compared with more conservative treatment options.^71^ As with many treatments, these outcomes are more positive when used in the early stages of disease, with up to 100% of hips surviving 3 years^69^ and up to 96% surviving 10 years in early-stage disease.^71^ More precisely, CD has shown positive results in osteonecrosis showing no collapse, a central lesion, and small size (combined necrotic angle <250°).^72^ These outcomes may prove even more beneficial when paired with grafts and cell-based therapy.

#### Vascularized and Nonvascularized Bone Grafting

Nonvascularized bone grafting involves the placement of bone graft material to provide structural support with the intent of reducing intraosseous pressure and preventing collapse in early stages of osteonecrosis. Vascularized bone grafting (VBG) also seeks to introduce increased blood supply. The graft is done by placing a nonvascularized cortical allograft from the ilium, tibia, or fibula,^73^ or a vascularized graft from the iliac crest, fibula, or greater trochanter^74^ into a core space created for the procedure or from a CD procedure. Nonvascularized bone grafting has shown moderate success, especially with smaller lesions, having a 55% to 87% success rate with a 2- to 9-year follow-up across several studies.^69^ VBG has shown a 5-year hip survival of 80% in precollapse lesion or 60% after 14 years in similar patients,^69^ with low conversion to THA.^75^ However, the benefits of VBG are primarily realized in smaller lesions without notable collapse.^76^ Ongoing research has evaluated synthetic scaffolds used with or without biofactors to enhance integration and bone growth. Numerous organic, inorganic, and biologic materials have been developed with promise, although no definitive solution has been identified.^77^

#### Adjunctive Therapy

Because osteonecrosis is thought to result from a deficiency of bone regeneration, use of stem cell treatments has been proposed to halt or reverse its pathogenesis. Studies have shown lower rates of radiographic progression and lower need for THA in patients treated with autologous stem cell transplants. In early studies, the combination of autologous stem cell transplant with CD showed a notable delay of an average of 10 years (up to 17 years) in time to collapse.^78^ In addition, cell therapy can be combined with other therapies such as CD and/or bone grafts and can potentially improve outcomes.^69^ A study has shown benefits of bone morphogeneticprotein (BMP) in addition to allograft and/or CD in improving bone formation and limiting the progression on osteonecrosis.^79^

#### Osteotomy

Osteotomy attempts to delay the progress of osteonecrosis by relieving weight-bearing on necrotic or prenecrotic areas to prevent collapse. To do this, weight-bearing osteonecrotic region is angled or rotated to place primary pressure on a non-necrotic area of the bone. Rotational (82% to 100% from 3 to 15 years) and angular (82% to 98% between 6 and 18 years) osteotomies of the femoral head have shown excellent success rates. However, future THA can become difficult if necessary because of persistent implant and altered anatomy.^69^

### Arthroplasty

#### Resurfacing

Resurfacing of the joints in question is the most minimal option for advanced osteonecrosis and involves replacing the articular surface with artificial materials to preserve natural anatomy. However, because of the complications from materials and possible contribution to osteonecrosis progression, resurfacing is no longer used as osteonecrosis treatment of the femoral head.^69^

#### Total Joint Arthroplasty

Joint arthroplasty is the only definitive cure for osteonecrosis available at this time; however, potential downsides require careful consideration. THAs are not a permanent solution, and although they may be beneficial early in older patients to reduce cumulative procedures, most patients with osteonecrosis are relatively young. Given this population, if the joint is replaced at diagnosis, the patient will likely need another arthroplasty or revision later in life. Recommendations for joint arthroplasty include advanced disease, continuing progression, and continuing provocative factors.^77^ Although patients who have a THA for osteonecrosis have more comorbidities and more complicated hospital stays than those having THA for osteoarthritis, long-term follow-up has shown similar outcome between the two groups for implant survival, osseointegration, and complications such as aseptic loosening.^80^ Other studies, however, have shown increased rates of sepsis,^81^ transfusion requirement, and hospital readmission in patients with osteonecrosis who underwent THA compared with OA patients. Recent analysis has shown improved outcomes, with >90% of osteonecrosis THAs surviving 4 to 7 years compared with 8 to 37% survival rates before 1990, possibly because of improved implants and materials used in the procedures.^69^ The literature is limited in examining etiology-based implant survival, but a study of patients with osteonecrosis secondary to alcohol consumption showed excellent long-term outcomes.^82^ It is also important to note that the study of patients with osteonecrosis requiring THA found that 46.6% of the patients would go on to require contralateral THA, especially if the contralateral hip had radiographic evidence of osteonecrosis at the time of the first THA, suggesting the need for a close follow-up.^83^

## Summary

Osteonecrosis continues to be a condition of widely variant etiologies, treatments, and developmental profiles. Because incidence continues to rise, increased understanding of the pathophysiology is necessary to promote developments of new treatments and corrective procedures. Although promising developments are being made in areas such as bone grafting and stem cell therapy, the field continues to lack an agreed-upon regimen to provide patients with osteonecrosis the greatest quality of life and delay their progression to debilitating injury, collapse, or joint arthroplasty. To more effectively understand this disease process, more data are needed. A national registry would be the most complete system to determine diagnostic and treatment directions. In the absence of such a coordinated effort, institutional registries and large cohort studies would help to make advances in this realm.

In the area of treatment, there are many potential avenues for improvement. Promising advancements in bone repair such as anabolic agents may play a role in promoting healing. In addition, more directed therapies for coincident conditions may reduce the secondary development of osteonecrosis from steroids and chemotherapy. With an expanded study of etiologies, prevention, and therapy, there is a reason to hope for advancements in reducing the burden of this disease.
